# The Clinical Utility of Automated Immature Granulocyte Measurement in the Early Diagnosis of Bacteremia

**DOI:** 10.7759/cureus.53660

**Published:** 2024-02-05

**Authors:** Lakshmipriya V, Kavitha K, Yogalakshmi E, Sridevi M

**Affiliations:** 1 Department of Pathology, Saveetha Medical College and Hospital, Saveetha Institute of Medical and Technical Sciences, Saveetha University, Chennai, IND

**Keywords:** complete blood count (cbc), bacteremia, biomarker, immature granulocytes, automated haematology analyzer

## Abstract

Background

Early diagnosis and prompt management are crucial for bacteremia and sepsis, as they have the potential to lead to septic shock and fatal outcomes. Bacteremia induces the recruitment of immature granulocytes (IGs) into the circulation, which indicates active bone marrow response. The goal of our present study is to determine the effectiveness of automated IG measurement as an alternate indicator for infection and also its clinical utility in predicting positive blood culture (BC) results.

Methods

We conducted a retrospective study including 100 BC-positive patients for whom complete blood count (CBC) and BC were done at the same time. Multiple hematological parameters including total white blood cell count (TWC), absolute neutrophil count (ANC), absolute lymphocyte count (ALC), IG count (IGC), and IG percentage (IG%) were obtained from the automated hematology analyzer, and IGC/TWC (IG ratio), IGC/ANC (immature-to-total neutrophil ratio), and ANC/ALC (neutrophil-to-lymphocyte ratio) were calculated using the primary data and compared with 100 uninfected normal individuals.

Results

The mean value of IG% and IGC between culture-positive and culture-negative groups were statistically significant (p-value < 0.05), suggesting that they are potential markers for bacteremia, and also the IG% was significantly higher in patients with positive BCs.

Conclusion

IG measurement is an easily accessible, cost-effective potential marker for screening bacteremia. Therefore, IGC and IG% could be incorporated as a part of the CBC report.

## Introduction

Bacteremia is characterized by the circulation of live bacteria or their toxins in the bloodstream. It occurs when bacteria manage to evade the body's immune system or when the immune response is inadequate in stopping bacterial proliferation due to inherent or acquired immune deficiencies. Bacteremia can progress to a severe condition known as bacterial sepsis, which is associated with significant organ dysfunction. Failure to diagnose and promptly address bacteremia and sepsis can lead to septic shock, multiple organ dysfunction syndrome, and, ultimately, a fatal outcome. Blood culture (BC) is the gold standard method for diagnosing bacteremia; however, it has a lengthy incubation period [1]. Therefore, other potential laboratory parameters generated on white cell analysis by automated hematology analyzers could be used in diagnosing bacteremia earlier. Quantifying leukocytes, especially neutrophils and their precursors, is important to predict infection because of their important role in acute inflammation and controlling bacterial infection [2].

In the past, the manual "band count" was a commonly employed marker for bacterial infection in pediatric practice. However, it posed challenges in terms of accurate measurement due to variability among hematologists. Over the recent years, automated hematology analyzers have seen significant technological advancements. These innovations have introduced features such as the automated immature granulocyte count (IGC) and immature granulocyte percentage (IG%), which are now accessible in modern hematology analyzers. These automated measures are regarded as highly reliable and precise indicators for early detection of sepsis [3]. Immature granulocytes (IGs) are the precursor of white blood cells, which include myelocytes, promyelocytes, and metamyelocytes. At present, automated IG measurements are still being evaluated for research purposes and not included in routine reporting. Monitoring these hematological parameters in suspicious cases of infection could help clinicians in early detection of bacteremia and sepsis, guide treatment decisions, reduce morbidity and mortality, and decrease health care costs. Thus, the goal of our present study is to assess the efficacy of automated IG measurement as an alternate marker for infection and also its clinical utility in predicting positive BC results.

## Materials and methods

This was a retrospective study conducted in our institute from January to June 2023. The study population included 100 patients of both sexes with age ≥ 18 years, who were clinically suspected of having an infection and, as part of their routine diagnostic evaluation, complete blood count (CBC) and BC tests were prescribed. Patients who were currently on antibiotics, those on steroid therapy, immunocompromised individuals, those with hematologic malignancy, and pregnant women were excluded from the study. CBC and BC screenings were done using samples obtained at the same time. CBC samples were collected by venipuncture in ethylenediaminetetraacetic acid (EDTA) vacutainer tubes and were analyzed using an automated hematology analyzer from the Sysmex XN series (Sysmex, Lincolnshire, IL) within 4 hours of collection. BC samples were obtained through venipuncture, with approximately 1 mL of blood collected into vials. These vials were then placed in the BACTEC system, and any samples identified as positive underwent further steps, including gram staining and subculture. Multiple hematological parameters including total white blood cell count (TWC), absolute neutrophil count (ANC), absolute lymphocyte count (ALC), IG%, and IGC were obtained from the automated hematology analyzer, and IG ratio, immature-to-total neutrophil ratio (IT ratio), and neutrophil-to-lymphocyte ratio (NLR) were calculated in the Microsoft Excel Spreadsheet (Microsoft Corp., Redmond, WA) using the primary data. The IG count included the sum of promyelocytes, myelocytes, and metamyelocytes. These hematological parameters were compared with 100 uninfected outpatient samples of both sexes with age ≥ 18 years that were selected randomly without available BC. Statistical analysis was conducted using SPSS statistical software (IBM Corp., Armonk, NY).

## Results

The study included 100 BC-positive individuals (56 males and 44 females) as a study group and 100 random BC-negative individuals (63 males and 37 females) as a comparison group. Among both BC-positive and BC-negative individuals, the common age group was 41 to 50 years, as shown in Table 1.

**Table 1:** Age distribution between blood culture positive and negative individuals

Age (years)	Blood culture positive (n=100)	Blood culture negative (n=100)
21-30	3	8
31-40	19	22
41-50	28	31
51-60	22	21
61-70	23	18
71-80	5	0

**Figure 1:**
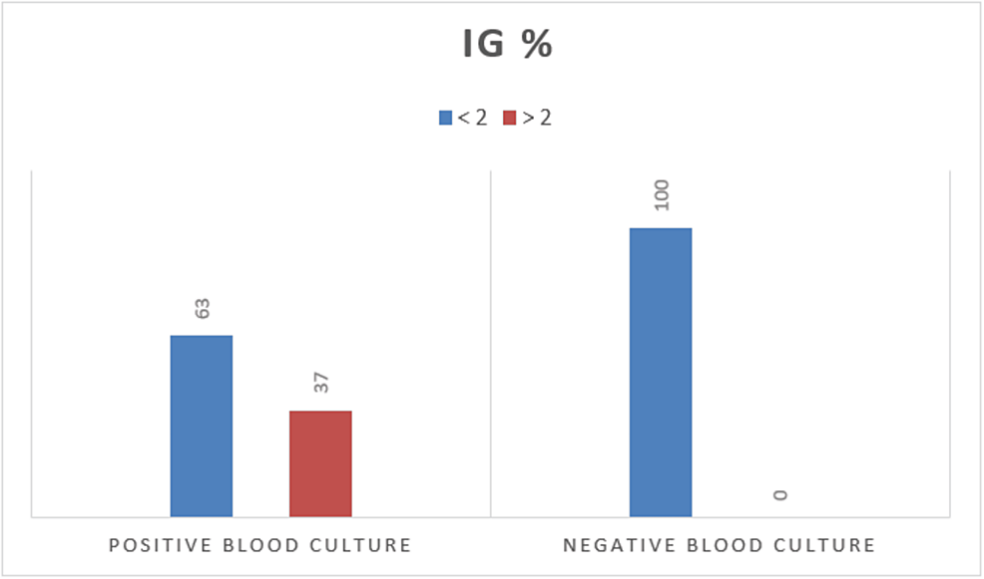
IG% in blood culture positive and negative individuals IG%, immature granulocyte percentage

The percentage of IG was notably elevated in patients with positive BC results. Among the 100 individuals who tested positive in the BC, 37 displayed IG% of 2% or higher, whereas none of the individuals with negative BC results exhibited IG% at or above 2% (Figure 1).

Table 2 provides a summary of the range of values, mean values with their respective 95% confidence intervals, and standard deviations for various hematological parameters, including TWC, ANC, IGC, IG%, NLR, IT, and IG ratios, within subgroups of patients with positive and negative BC results. The means of IGC, IG%, NLR, IG ratio, IT ratio, TWC, and ANC among the culture-positive patients were high and also statistically significant (p-value < 0.05) when compared to culture-negative patients (Table 2), suggesting that they are potential markers for bacteremia. The statistical analysis was conducted using an unpaired t-test.

**Table 2:** Comparison of hematological parameters between blood culture positive and negative individuals TWC, total white blood cell count; ANC, absolute neutrophil count; ALC, absolute lymphocyte count; IG%, immature granulocyte percentage; IGC, immature granulocyte count; NLR, neutrophil-to-lymphocyte ratio; IT ratio, immature-to-total neutrophil ratio; IG ratio, immature granulocyte ratio

Hematological parameters	Positive blood culture (n=100)					Negative blood culture (n=100)					p-value (<0.05)
	Min	Max	Mean	SD	95% CI	Min	Max	Mean	SD	95% CI	
TWC (×10^3^/cumm)	2.69	44.79	14.21	8.14	12.592-15.822	5.15	14.4	8.534	1.64	8.209-8.859	<0.0001
ANC (×10^3^/cumm)	1.31	42.24	11.73	7.81	10.178-13.278	2.68	9.98	5.225	1.35	4.958-5.492	<0.0001
ALC (×10^3^/cumm)	0.31	4.35	1.641	0.88	1.466-1.816	0.57	3.57	2.024	0.57	1.911-2.137	0.0003
IG%	0	12.2	1.935	2.1	1.517-2.352	0	1.3	0.386	0.27	0.333-0.438	<0.0001
IGC (×10^3^/cumm)	0	4.05	0.345	0.58	0.230-0.459	0	0.15	0.036	0.03	0.031-0.042	<0.0001
NLR	1.16	51.02	9.425	8.61	7.717-11.133	1.08	4.05	2.87	1.49	2.575-3.164	<0.0001
IT ratio	0	67.84	3.311	8.67	1.591-5.031	0	3.55	0.732	0.58	0.617-0.847	0.0034
IG ratio	0	49.88	2.416	5.74	1.277-3.554	0	1.5	0.429	0.29	0.371-0.488	0.0007

**Figure 2:**
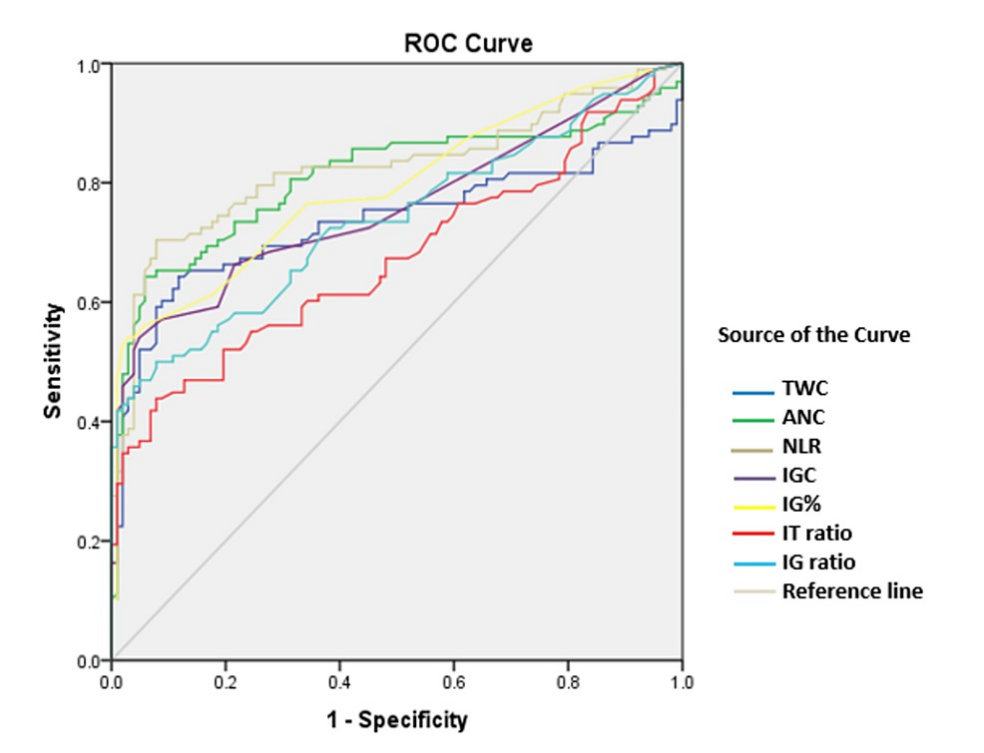
Comparison of ROC curves among all studied parameters ROC, receiver operating characteristic; TWC, total white blood cell count; ANC, absolute neutrophil count; NLR, neutrophil-to-lymphocyte ratio; IGC, immature granulocyte count; IG%, immature granulocyte percentage; IT ratio, immature-to-total neutrophil ratio; IG ratio, immature granulocyte ratio

The diagnostic efficacy of the hematological parameters mentioned above in predicting infection was assessed using receiver operating characteristic (ROC) curves (Figure 2). ROC plots displayed sensitivity versus 1-specificity, such that areas under the curve (AUC) generated varied from 0.7 to 0.8, with higher values indicating increased discriminatory ability.

All parameters studied had an AUC between 0.7 and 0.8 (IT ratio < IG ratio < TWC < IGC < IG% < ANC < NLR), as seen in Table 3, indicating that they are markers of bacteremia.

**Table 3:** Area under the curve for the hematological parameters TWC, total white blood cell count; ANC, absolute neutrophil count; NLR, neutrophil-to-lymphocyte ratio; IGC, immature granulocyte count; IG%, immature granulocyte percentage; IT ratio, immature-to-total neutrophil ratio; IG ratio, immature granulocyte ratio

Parameter	Area under the curve
TWC	0.73
ANC	0.8
NLR	0.82
IGC	0.75
IG%	0.79
IT ratio	0.67
IG ratio	0.73

## Discussion

Every year, millions of patients succumb to sepsis, a condition that inflicts severe harm upon human well-being. Hence, timely identification and suitable intervention play a crucial role in improving the prognosis of septic patients [4]. When faced with systemic inflammation, injury, or stress, the innate immune system employs lymphocytopenia and neutrophilia as its physiological responses [5]. Numerous investigations have explored the clinical utility of both quantitative and qualitative alterations in leukocytes for predicting infections in both pediatric and adult populations. Features such as toxic granules, Dohle bodies, and cytoplasmic vacuoles, which were observed microscopically in peripheral smear examination, exhibited a high level of sensitivity but lacked specificity when it came to predicting infections. The parameters associated with leukocytes appear to have limitations in terms of both sensitivity and specificity in predicting infections. This is because increases in IG, neutrophil, and band counts are not exclusive to infections; they can also manifest in various other conditions including chronic inflammatory diseases, neoplastic disorders, acute hemorrhage, hemolysis, tissue damage or necrosis, seizures, metabolic abnormalities, use of specific medications, and myeloproliferative neoplasms [2]. However, many studies tried to combine IG measurement with WBC, ANC, or other markers to increase the accurate prediction of microbial infection and identified IGC and IG% as innovative indicators for sepsis.

The quantification of IG is traditionally done through manual microscopy, which is a time-consuming and labor-intensive process and suffers from significant variability among different observers [6]. In our evaluation, we assessed IG as determined by the Sysmex automated blood counter as a potential marker for predicting infection. In automated analyzers, IG is measured using flow cytometry within the white blood cell differential fluorescence (WDF) channel. This measurement relies on assessing cell granularity (side scatter) and nucleic acid content (side fluorescence). The automated IG count offers distinct advantages over the manual count, demonstrating superior specificity, sensitivity, and precision. This improvement is particularly evident when compared to manual counting, which can vary based on the number of cells counted and its clinical application [7].

A study conducted by Senthilnayagam et al. included adults, children, infants, and neonates as their study population in which IGC of 0.03 × 103/µL and IG% of 0.5% offered a sensitivity of 86.3% and 92.2%, respectively [3]. The study conducted by Ayres et al. showed that IG% < 2.0% is helpful in the exclusion of sepsis diagnosis before BC results, with a very high specificity (90.9%) [8]. Similarly, a study conducted by van der Geest et al. found that the median IG% was 1.8% in patients with positive BC results, which was indicative of infection, while it was 0.3% in the non-infectious group [9]. In our present study, none of the individuals with negative BC results had IG% of 2% or more with a sensitivity of 63%. According to Bhansaly et al., IGC and IG% emerged as the earliest biomarkers with substantial discriminatory potential with AUC values of 0.81 and 0.82, respectively, when compared to other biomarkers, including procalcitonin and blood lactate [10]. Similarly, receiver operating characteristic curves in our study showed that IGC and IG% are better predictors of infection with AUC of 0.75 and 0.79, respectively.

Jayasekara et al.’s study included 42 neonatal samples analyzed for manual IT ratio, of whom 21 had an IT ratio of ≥0.2 (positive), among whom 15 were finally diagnosed with sepsis (true positive) and six were negative (false positive), and the sensitivity and negative predictive value for the manual IT ratio were 93.8% and 95.2%, respectively [11]. Iddles et al.’s study results showed that a large proportion of septic patients had raised ratios, particularly IT ratios, with one as high as 37.5, and in negative control group, some showed increased value but not more than 3 [12]. Our study showed that the maximum value for IT ratio was 67.84 and that for IG ratio was 49.88 in BC-positive patients.

Limitations of the study

This is a single-center retrospective study conducted in a limited sample size with variable clinical heterogeneity. We did not include the comorbidities of the individuals included in the study. Also, we did not establish a correlation between the hematological parameters and the spectrum of bacterial species isolated from BC.

## Conclusions

IG measurement is an easily accessible cost-effective potential marker for screening bacteremia, thus facilitating early intervention and leading to greater possibilities of recovery. Along with additional laboratory parameters, we can also monitor the response to treatment, which helps clinicians in assessing the effectiveness of therapy. Therefore, IGC and IG% could be incorporated as a part of CBC. To further establish the robustness and generalizability of these findings, future research endeavors should consider larger, prospective, and multi-center studies.
